# Supplementary material for: The antibiotic bedaquiline activates host macrophage innate immune resistance to bacterial infection
Source: eLife. 2020 May 4;9:e55692. doi: 10.7554/eLife.55692 (PMC7200153; doi:10.7554/eLife.55692)
Supplement: Supplementary file 2. [file elife-55692-supp2.docx]

**Oligonucleotide sequences**

The table lists all primers used in this study.

| **Gene** | **Forward** | **Reverse** |
| --- | --- | --- |
| *atpe* (*Mycobacterium tuberculosis*) | 5-TCGTGTTCATCCTGATCTCCA-3 | 5-GACAATCGCGCTCACTTCAC-3) |
| *atp6v0a1* (*Homo sapiens*) | 5-TCACCGACCTTGACTCCATCCA-3 | 5-GGAGTCTGGTTTGTCTGCATCC-3 |
| fabp5 (*Homo sapiens*) | 5-GGAAGGAGAGCACGATAACAAGA-3 | 5- GGTGGCATTGTTCATGACACA-3 |
| *hERG* (*Homo sapiens*) | 5-GGGCTCCATCGAGATCCT-3 | 5-AGGCCTTGCATACAGGTTCA-3 |
| *mcoln1* (*Homo sapiens*) | 5-CGGACTGCTATACCTTCAGCGT-3 | 5-GGTGCTTACACTCCTGGATGTG-3 |
| *rpl24* (*Homo sapiens*) | 5-CAAAAGAAAAGAACCCGCCGA-3 | 5-TCGAAACTGGGGAACCATGA-3 |
| *scarb1* (*Homo sapiens*) | 5-CTTGTTTCTCTCCCATCCTCA- | 5-GAGTGTGCCTCCTGGTTAG-3 |
| *trpm2* (*Homo sapiens*) | 5-ACGTGCTCATGGTGGACTTC-3 | 5-AGGGTCATAGAAGAGCTGCC-3 |
| *dnase2* (*Homo sapiens*) | 5-CCTGCTCTACAATGACCAACCG-3 | 5-GTACACTGTGGACCAGCCAGAA-3 |
| *gga2* (*Homo sapiens*) | 5-CAGTGATGCTCCTGTTACAGGC-3 | 5- GGTCCAGCAAATTCCTGTCTGC-3 |
| *glb1* (*Homo sapiens*) | 5-CACTCCACAATCAAGACCGAAGC-3 | 5-CTGTGCTGCATAGGGTGAGTTG-3 |
| *igf2r* (*Homo sapiens*) | 5-TTGAGTGGCGAACGCAGTATGC-3 | 5-CAGTGATGGCTTCCCAGTTGTC-3 |
| *lgmn* (*Homo sapiens*) | 5-CCTGAAGATGGAGGCAAGCACT-3 | 5-GTTCGTCAGGAATCCCATTGCG-3 |
| *neu1* (*Homo sapiens*) | 5-TCCAAGGCTGAGAACGACTTCG-3 | 5-TCAGCAAAGGCGAGAAGAGTGC-3 |
| *npc1* (*Homo sapiens*) | 5- TCTCTTTGCGGGATTGGCAGTC-3 | 5-CGCTTGTTCCATCTTCAGCACC-3 |
